# Cluster Headache Impact Questionnaire (CHIQ) – a short measure of cluster headache related disability

**DOI:** 10.1186/s10194-022-01406-y

**Published:** 2022-03-18

**Authors:** Katharina Kamm, Andreas Straube, Ruth Ruscheweyh

**Affiliations:** Department of Neurology, University Hospital, Ludwig-Maximilians-University (LMU), Munich, Germany

**Keywords:** Cluster headache, Disability, Questionnaire, Patient-reported outcome measure

## Abstract

**Background:**

Cluster headache (CH) is a severe, highly disabling primary headache disorder. However, there is little research on CH-related disability, and most of it is based on non CH-specific questionnaires. The aim of this study was to develop a short, CH-specific disability questionnaire.

**Methods:**

The 8-item Cluster Headache Impact Questionnaire (CHIQ) was developed based on a literature review and patient and expert interviews. The questionnaire was tested in 254 CH patients (171 males; 47.5 ± 11.4 years; 111 chronic CH, 85 active episodic CH, 52 episodic CH in remission) from our tertiary headache center or from a German support group.

**Results:**

Reliability and validity of the CHIQ was evaluated in active episodic and chronic CH patients (*n* = 196). Internal consistency (Cronbach’s α = 0.88) and test-retest reliability (ICC 0.91, *n* = 41) were good. Factor analysis identified a single factor. Convergent validity was shown by significant correlations with the Headache Impact Test (HIT-6, *r* = 0.58, *p* < 0.001), subscales of the depression, anxiety and stress scales (DASS, *r* = 0.46–0.62; *p* < 0.001) and with CH attack frequency (*r* = 0.41; *p* < 0.001). CHIQ scores significantly differentiated between chronic CH (25.8 ± 6.5), active episodic CH (23.3 ± 6.9) and episodic CH patients in remission (13.6 ± 11.9, *p* < 0.05 for all 3 comparisons).

**Conclusions:**

The CHIQ is a short, reliable, valid, and easy to administer measure of CH-related disability, which makes it a useful tool for clinical use and research.

**Supplementary Information:**

The online version contains supplementary material available at 10.1186/s10194-022-01406-y.

## Background

Cluster headache (CH) is defined by recurrent, short-lasting, unilateral and severe headache attacks accompanied by restlessness and ipsilateral cranio-autonomic symptoms (CAS) like lacrimation, conjunctival injection, rhinorrhea or nasal congestion. Up to eight headache attacks/ day can occur during active episodes, typically separated by headache-free remission periods. Opposed to this episodic course of disease affecting 80% of patients, chronic CH patients suffer from attacks without remission periods longer than three months/ year [1]. Active phases of the disease and headache attacks often show circannual and circadian rhythmicity. Nocturnal attacks are common [2].

Although the excruciating character of CH attacks is well known, disability associated with the disorder has only recently come to focus. Due to the lack of CH-specific questionnaires, generic or migraine-specific questionnaires like the Headache Impact Test™ (HIT-6™), Migraine Disability Assessment (MIDAS), SF-12v2® Health Survey (SF-12v2®) or Migraine Specific Quality of Life Questionnaire Version 2.1 (MSQ v2.1) have mostly been used [3]. Although these studies have shown reduced quality of life and substantial disability in CH patients, it has been questioned whether CH-specific impairment is adequately captured or rather underestimated by these instruments [4]. One disadvantage of generic questionnaires is that they don’t evaluate CH-specific characteristics like frequent daily or nocturnal attacks or agitation. Further, they use timeframes of weeks to months that may not be appropriate in CH patients due to rapid changes in attack frequency. To overcome these problems, two CH-specific questionnaires concerning quality of life (QoL) and psychosocial factors have been developed and validated, the 28-item Cluster Headache Quality of Life Scale (CHQ) and the 36-item Cluster Headache Scales (CHS), with the latter including an 11-item disability subscale [5, 6].

Nevertheless, to date, a decidedly short, CH-specific disability questionnaire is still lacking. Such an instrument would be extremely useful both for following the course of CH patients in daily clinical practice and for use as a patient-reported outcome measure (PROM) in clinical trials, since subjective ratings have become increasingly important outcome parameters [7–9].

The objective of this study was to develop a short questionnaire to specifically assess the current impact of CH on daily life and to demonstrate reliability and validity of this instrument.

## Methods

### Development of the questionnaire

After an in-depth review of the general CH literature and the literature on CH-related disability (see below) and general headache-related disability, and extensive discussions between the authors, a first (11 item) version of the questionnaire was drafted. Special care was taken to address CH-specific problems like sleep disturbance by attacks at night, unpredictability of attacks, between-attack disability and self-injurious behavior, while keeping the questionnaire short. This first version was discussed with a number of German headache experts, resulting in elimination of 3 items, and in some rewording. Face validity of this second version was tested in ten CH patients (completing the questionnaire followed by a personal interview, assessing comprehensibility of instructions and relevance regarding the impact of CH on patients’ lives), and some minor rewording was performed.

The resulting third version of the Cluster Headache Impact Questionnaire (CHIQ) consisted of eight items to be rated on a six-point Likert scale from “never” (score 0) to “always” (score 5) (Additional file 1), resulting in a sum score ranging from 0 to 40. Two questions ask for CH-associated limitations in work, household, family and social life and recreational activities. Four questions assess concentration difficulties, irritability, fatigue due to nocturnal attacks and limitations of daily life activities due to poor predictability of headache attacks. Further, two questions ask for self-injurious behavior and the impression of being a burden to the patient’s social environment. CH attack frequency and severity often change rapidly, and CH impact likely follows these changes to a certain extent. To allow assessment of the *current* impact of CH, we therefore referred all questions to the last week. Two extra questions (not forming part of the CHIQ score) assess attack frequency and acute medication intake during the last week.

For the purpose of publication, the CHIQ was translated from the original German version to English using a standard forward-backward translation procedure [10]. 

We compared results of the CHIQ with the results of other questionnaires that are often used to assess the impact of headache on disability and quality of life, and of headache-related cofactors such as depression, anxiety and stress [4].

### Headache impact test (HIT-6)

The Headache Impact Test (HIT-6) is a six item self-report questionnaire developed to assess the impact of headaches on daily activities in a general headache population [11]. Questions concerning impact on daily activities, the desire to lie down, headache-related fatigue, irritability, concentration difficulties and frequency of severe pain are answered on a five-item scale ranging from “never” to “always”. Three of the questions are presented without a timeframe, three refer to the past four weeks. Items are scored from 6 to 13, resulting in a total score of 36 to 78. The questionnaire shows good reliability and validity [12]. The HIT-6 was chosen because it is one of the most used headache-related disability questionnaire, it is similar to the CHIQ due to its brevity and disability is rated on a scale, as opposed to counting days with disability (as opposed to the MIDAS).

### Depression, anxiety and stress scale – 21 items (DASS-21)

The DASS is a self-report 21-item questionnaire assessing the emotional states of depression (DASS-D), anxiety (DASS-A) and stress (DASS-S) over the last week (subscores of seven items each). Each item is rated on a four-point scale ranging from 0 (“did not apply to me at all”) to 3 (“applied to me very much”), resulting in subscores ranging from 0 to 21. The DASS is a widely used in headache research, easy to administer, freely available questionnaire with good reliability and validity [13, 14].

### Short form-12 health survey (SF-12v2)

The SF-12v2 is one of the most used health-related quality of life questionnaires, consisting of 12 items. It is a generic measure with good validity and reliability [15]. It measures eight health domains comprising physical functioning (PF), general health (GH), role physical (RP), bodily pain (BP), social functioning (SF), vitality (VT), mental health (MH) and role emotional (RE). Items are answered on Likert scales, raw scores are summed up and transformed to a scale from 0 to 100. Two summary scores – mental component score (MCS) and physical component score (PCS) – are obtained and were considered in the present analysis. The PCS is a measure of physical functioning and the MCS evaluates general mental well-being and absence of psychological distress. Higher scores indicate better quality of life. SF-12v2 was analyzed using Quality Metric Health Outcomes Scoring (https://www.qualitymetric.com/). The SF-12 was preferred over the SF-36 because of its brevity in order to minimize missing data.

### Participants and study procedures

The study was approved by the local ethics committee of the University of Munich (20–738) and was conducted in accordance with the Declaration of Helsinki.

Between October 2020 and February 2021, patients with episodic or chronic CH according to the ICHD-3 criteria [1] were recruited at the Upper Bavarian Headache Center at the LMU Hospital Munich and via a German support group (federal association of cluster headache support groups (CSG)). Patients were invited personally or via mail or e-mail and they could choose to participate either using an online survey or a paper-based questionnaire.

The online survey was conducted using RedCap (Research Electronic Data Capture) [16, 17]. After obtaining informed consent, participants were asked to create a personal code in order to match first and second survey participation (see below) and to screen for duplicate participation that were excluded from data set (*n* = 27). The survey comprised five questionnaires: the CHIQ, a customized questionnaire assessing demographics, medical history and clinical characteristics of CH (including headache characteristics, attack abortive and prophylactic treatment), the HIT-6, the DASS and the SF-12v2. Participants also rated suitability of the CHIQ and the HIT-6 to assess CH-related disability on a 5-point Likert scale. After completing the questionnaires, participants were asked to take part in a follow-up survey. If they consented, they received an invitation link or the paper-based follow-up questionnaire 14 days later. The follow-up survey comprised the CHIQ, a short customized questionnaire assessing the current status of CH and treatment, HIT-6, DASS and SF12v2.

Subjects were included in the analysis if they were ≥ 18 years old and had an episodic or chronic CH diagnosis, as confirmed by review of the clinical characteristics section of the questionnaire according to ICHD-3 criteria [1]. Subjects with missing data in the CHIQ were excluded. Incomplete HIT-6, DASS or SF-12v2 questionnaires weren’t evaluated for the respective participant. Single missing items in demographics, clinical characteristics and treatment questionnaire were allowed.

**Fig. 1:**
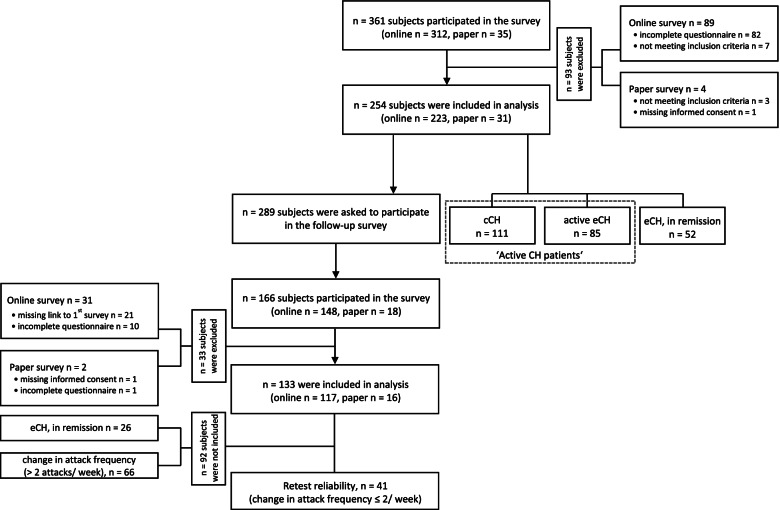
Participant disposition. For analysis of reliability and validity, chronic and active episodic CH patients, named ‘active CH patients’ (*n* = 196), were evaluated. In this figure, this group is highlighted by the broken fringe. After 14 days, patients were invited to take part in a second survey to evaluate test-retest reliability. Only participants with active CH at both surveys and a change in attack frequency ≤ 2 attacks per week (from first to follow-up survey) were included in the analysis of test-retest reliability (*n* = 41). cCH, chronic cluster headache; eCH, episodic cluster headache

Participant disposition is shown in Fig. 1. Finally, data was available from 254 participants for analysis of the first questionnaire, and data was available from 133 participants for analysis of the follow-up questionnaire. This short-term follow-up was aimed to address stability of the items and the questionnaire, therefore only participants with active CH at both surveys and a stable attack frequency (a change ≤2 attacks per week from first to follow-up survey) were included in the analysis of test-retest reliability (*n* = 41).

### Statistical analysis

Demographics and CH characteristics are presented as descriptive statistics (mean ± SD or numbers and percentages of patients). The Shapiro-Wilk test was used to evaluate normality of data distribution.

To identify factors underlying the CHIQ, exploratory factor analysis (oblimin principal axes factor analysis, PFA) was performed after confirmation of suitability using the Kaiser-Meyer-Olkin (KMO) criterion and Bartlett test. We had no a priori hypothesis about the factor structure of the questionnaire.

Item statistics comprising item difficulty and item-scale correlations were assessed. For internal consistency, Cronbach’s alpha was calculated. A Cronbach’s alpha > 0.80 was accepted as good [18, 19].

Test-retest reliability was assessed using intraclass correlation coefficients (ICCs, two-way mixed effect model with absolute agreement for single measures) and Wilcoxon’s test for comparison of mean values [20].

Convergent validity between the CHIQ, CH characteristics and the results of other questionnaires were assessed using Spearman correlations, and the same correlations were later also calculated with the HIT-6 score.

Group differences between episodic (active and in remission) and chronic CH patients were assessed using a Kruskal-Wallis-ANOVA followed by Wilcoxon tests with Bonferroni correction for 3 comparisons.

Statistical analysis was performed using SPSS Statistics 26 (IBM Corp., Armonk, NY, USA). Significance was accepted at *p* < 0.05 (two-tailed).

## Results

254 subjects were included in the analysis (m = 171; 47.5 ± 11.4 years; 111 chronic cluster headache (cCH), 85 active episodic cluster headache (eCH), 52 eCH in remission). To demonstrate reliability and validity of the CHIQ in patients currently suffering from attacks, the main analysis was based on chronic and active episodic CH patients (‘active patients’, *n* = 196, m = 128, 47.2 ± 11.6 years). This group indicated 15.2 ± 13.8 attacks and 13.5 ± 14.2 acute medication uses in the last week. Additional demographics and clinical characteristics are listed in Table 1.

**Table 1:** Clinical characteristics of study sample

	cCH	active eCH	‘active CH‘	eCH, in remission
n (male)	111 (58)	85 (70)	196 (128)	52 (39)
Gender ratio (male: female)	1.09: 1	4.67: 1	1.88: 1	1.33: 1
Age (years)	48.06 ± 11.55	46.09 ± 11.73	47.21 ± 11.64	48.65 ± 10.87
Age at onset (years)	32.55 ± 13.14	29.25 ± 11.54	31.13 ± 12.55	27.44 ± 11.23
Disease duration (years)	15.52 ± 9.46	17.19 ± 11.68	16.24 ± 10.49	21.21 ± 10.30
Attack frequency (attacks in last week)	16.50 ± 13.66	13.42 ± 13.75	15.16 ± 13.75	0
Nocturnal attacks, n (%)	102 (91.9%)	79 (92.9%)	181 (92.3%)	43 (82.7%)
Headache intensity [0–10]	7.55 ± 2.10	7.80 ± 1.73	7.66 ± 1.95	7.95 ± 2.06
Number of cranial autonomic symptoms	4.74 ± 1.48	4.22 ± 1.60	4.52 ± 1.57	4.33 ± 1.70
Restlessness during attacks, n (%)	106 (95.5%)	83 (97.6%)	189 (96.4%)	50 (96.2%)
Typical episode duration (weeks)	–	12.00 ± 8.82	–	11.94 ± 8.10
Current acute medication, n (%)	105 (94.6%)	77 (90.6%)	182 (92.9%)	–
Acute medication uses (in last week)	14.25 ± 13.70	12.51 ± 14.80	13.49 ± 14.18	–
Current preventive medication, n (%)	76 (68.5%)	44 (51.8%)	120 (61.2%)	–
Other medical condition, n (%)	61 (55.0%)	35 (41.2%)	96 (49.0%)	16 (30.8%)
Current smoking, n (%)	65 (58.6%)	42 (49.4%)	107 (54.6%)	26 (50.0%)
Current alcohol consumption, n (%)	12 (10.8%)	27 (31.8%)	39 (19.9%)	19 (36.5%)

Data are shown as mean ± standard or number (percentage) of patients. CH, cluster headache; cCH, chronic cluster headache; eCH, episodic cluster headache

### Factor analysis

Data were suitable for factor analysis according to the KMO criterion (0.88) and Bartlett test (*x* ^2^(28) = 772.07, *p* < 0.001). Inspection of the scree plot and eigenvalues after principal axes factor analysis with oblimin rotation revealed one factor, accounting for 55.67% of the variance. Factor loadings were meaningful for all items (0.56 to 0.81, Table 2).

**Table 2:** Item and factor analysis and test-retest correlation of the CHIQ

	Mean (SD)	Item difficulty	Corrected item-scale correlation (with item deleted)	Cronbach’s α (with item deleted)	Factor loading	Intraclass Correlation	95% Confidence Interval	
							Lower Bound	Upper Bound
**CHIQ1**	3.37 (1.09)	67.4	0.72	0.86	0.79	0.82	0.67	0.91
**CHIQ2**	3.40 (1.03)	68.0	0.74	0.86	0.81	0.79	0.60	0.89
**CHIQ3**	3.51 (1.07)	70.2	0.66	0.86	0.71	0.81	0.65	0.90
**CHIQ4**	3.35 (1.01)	67.0	0.61	0.87	0.64	0.81	0.65	0.90
**CHIQ5**	3.19 (1.27)	63.8	0.67	0.86	0.73	0.83	0.67	0.91
**CHIQ6**	3.35 (1.08)	67.0	0.67	0.86	0.70	0.73	0.50	0.86
**CHIQ7**	1.61 (1.31)	32.2	0.53	0.88	0.56	0.91	0.83	0.95
**CHIQ8**	2.94 (1.25)	58.8	0.63	0.86	0.67	0.88	0.78	0.94
**CHIQ score**						0.91	0.83	0.95

This analysis was performed in the group of ‘active CH’ patients (*n* = 196). Each item showed good item analysis as well as meaningful factor loadings. Intraclass correlation coefficients (ICCs) of single CHIQ items and the CHIQ score are given

### Item and scale analysis

Results of the item analysis are shown in Table 2. Item difficulty was within the desired range (20–80%) and corrected item-scale correlations were good (> 0.5) [21]. Internal consistency of the CHIQ was good with Cronbach’s α = 0.88. Cronbach’s α did not increase after deletion of any of the items.

**Fig. 2:**
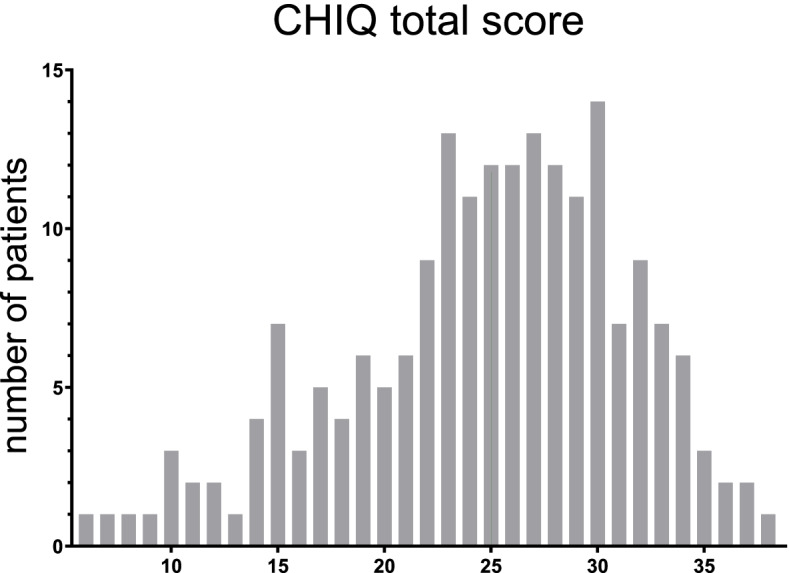
Histogram of CHIQ scores in ‘active CH’ patients (*n* = 196)

The average CHIQ score was 24.7 ± 6.8 (range 2–39, possible range 0–40) in active patients. The histogram showed a slightly left-skewed distribution (Fig. 2) but no ceiling or bottom effects [22]. Accordingly, the Shapiro-Wilk test revealed a significant deviation from normality (*p* < 0.05).

### Test-retest reliability

CH attack frequency often changes rapidly, and CH impact likely follows these changes to a certain extent. To assess test-retest reliability, we therefore selected those active patients who had similar attack frequencies at test and retest (i.e. a difference of 2 attacks per week or less). 41 patients fulfilled these criteria (15 eCH, m = 24, 47.5 ± 12.1 years, test-retest interval 24.5 ± 18.5 days). Average CHIQ values of the test (24.56 ± 6.5) and retest (23.71 ± 7.61) were not significantly different (Z = − 1.56, *p* = 0.12).

Test-retest reliability was also good (ICC = 0.91). Test-retest correlations for single items (ICCs) were between 0.73 and 0.91 (Table 2).

### Convergent validity

Convergent validity of the CHIQ was assessed by evaluating correlations with the HIT-6, the DASS and relevant subscales of the SF12v2 (Table 3). According to Cohen’s graduation [23], significant large positive correlations were found with the HIT-6 (*ρ* = 0.58, *p* < 0.001), DASS depression and stress subscales (*ρ* = 0.62 and 0.54, both *p* < 0.001) and significant large negative correlations were found with the SF12v2 MCS (*ρ* = − 0.51, *p* < 0.001) and SF12v2 PCS (*ρ* = − 0.49, *p* < 0.001). Significant correlations of moderate size were found with attack frequency (0.41, *p* < 0.001), acute medication frequency (0.37, *p* < 0.001), the DASS anxiety subscale (0.46, *p* < 0.001) and the CH pain AUC (0.44, *p* < 0.001).

**Table 3:** Mean values and correlations between CHIQ and HIT-6 scores, CH characteristics and other disability or QoL measures in ‘active CH’ patients (*n* = 196)

	Mean ± SD	Correlation with CHIQ	Correlation with HIT-6
**Attack frequency** (in last week)	15.16 ± 13.75	0.41, *p* < 0.001	0.21, *p* = 0.003
**Acute medication frequency** (in last week)	13.49 ± 14.18	0.37, *p* < 0.001	0.23, *p* = 0.001
**Cluster headache pain AUC** (in last week)	4810.63 ± 6794.37	0.44, *p* < 0.001	0.31, *p* < 0.001
**CHIQ**	24.73 ± 6.76	–	0.58, *p* < 0.001
**HIT-6**	63.14 ± 6.41	0.58, *p* < 0.001	–
**DASS-D (depression)**	9.14 ± 5.30	0.62, *p* < 0.001	0.61, *p* < 0.001
**DASS-A (anxiety)**	6.21 ± 4.62	0.46, *p* < 0.001	0.54, *p* < 0.001
**DASS-S (stress)**	10.15 ± 5.20	0.54, *p* < 0.001	0.58, *p* < 0.001
**SF12v2 PCS**	41.78 ± 8.69	−0.49, *p* < 0.001	−0.43, *p* < 0.001
**SF12v2 MCS**	37.13 ± 10.21	− 0.51, *p* < 0.001	−0.54, *p* < 0.001

Spearman correlations are given. CH pain AUC was calculated by multiplication of number of attacks in last week, attack duration in minutes and attack intensity. CHIQ, Cluster Headache Impact Questionnaire; HIT-6, Headache Impact Test; DASS, Depression, anxiety and stress scale; SF12v2, Short Form-12 Health Survey; PCS, physical component score; MCS, mental component score; CH cluster headache

### Group differences

**Fig. 3:**
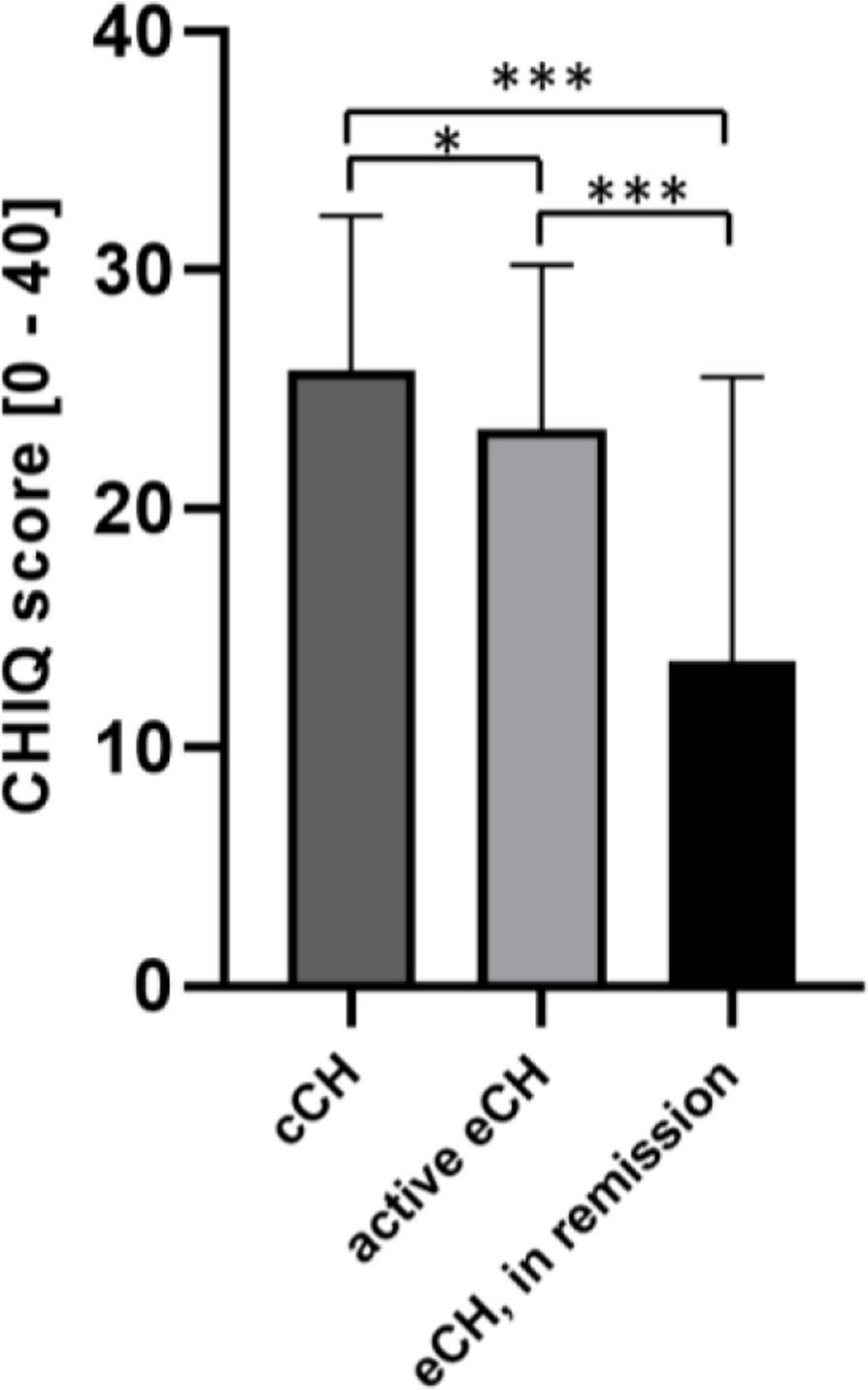
Distribution of CHIQ scores in CH patients. CHIQ total scores in cCH (*n* = 111), active eCH (*n* = 85) and eCH in remission (*n* = 52) patients. Chronic CH patients showed significantly higher CHIQ total scores compared to eCH patients, regardless of CH activity. Also, active eCH patients showed significantly higher scores compared to eCH patients in remission. *, *p* < 0.05, ***, *p* < 0.001. CHIQ, Cluster Headache Impact Questionnaire; CH, cluster headache; cCH, chronic cluster headache; eCH, episodic cluster headache

Average CHIQ scores were 23.3 ± 6.9 in active eCH patients (*n* = 85), 25.8 ± 6.5 in cCH patients (*n* = 111) and 13.6 ± 11.9 in eCH patients in remission (*n* = 52). Kruskal-Wallis ANOVA indicated significant group differences (H [2] = 41.2, *p* < 0.001). Posthoc tests with Bonferroni correction revealed significant differences between all three groups (*p* = 0.035 for active eCH vs. cCH, *p* < 0.001 for both comparisons with eCH in remission, Fig. 3).

### Comparison between CHIQ and HIT-6 for assessment of CH-related disability

Patients rated suitability of the CHIQ and the HIT-6 to assess CH-related disability on a 5-point Likert scale from 1 = “very well” to 5 = “not at all”. Ratings were significantly better for the CHIQ (2.3 ± 0.8) compared to the HIT-6 (2.4 ± 0.8, *n* = 185, Wilcoxon test Z = − 2.6, *p* = 0.011).

Regarding correlations with CH characteristics and other questionnaires, the CHIQ showed medium sized correlations with attack and acute medication frequency, while the respective correlations with the HIT-6 were weak, according to Cohen’s graduation [23]. In contrast, correlations with the DASS and SF12v2 were similar for the CHIQ and the HIT-6 (showing medium to large effect sizes, Table 3).

Similar to the CHIQ, Kruskal-Wallis ANOVA detected significant group differences for the HIT-6 scores (cCH: 63.78 ± 6.83, *n* = 106, active eCH: 62.28 ± 5.72, *n* = 79, eCH in remission: 52.67 ± 6.44, *n* = 49, H [2] = 70.8, *p* < 0.001) and posthoc tests with Bonferroni correction revealed significant differences between eCH in remission and both, cCH and active eCH (both *p* < 0.001). Different from CHIQ scores, HIT-6 scores were not significantly different between active eCH and cCH (*p* = 0.192).

### Preliminary CHIQ grading

The vast majority of our active CH patient group (76.4%) fell into HIT-6 grade 4, limiting usefulness of HIT-6 grades for clinical discrimination in this patient group. We therefore tentatively graded CHIQ scores according to quartiles, resulting in the following grades: grade 1 (CHIQ score 0 to 21, 26.5% of the patients), grade 2 (CHIQ score 22–25, 23.0%), grade 3 (CHIQ score 26–29, 24.5%) and grade 4 (CHIQ score 30–40, 26.0%). When attack and acute medication frequency, CH pain AUC, HIT-6 scores and DASS scores were plotted against CHIQ scores, monotonous ascending relations were verified, and ANOVA corroborated significant differences between CHIQ grades for these parameters (Supplementary Fig. 1). In eCH patients in remission, 70.7% corresponded to CHIQ grade 1 (and 12,1%, 8.6% and 8.6% to grades 2, 3 and 4, respectively).

## Discussion

Main result of the present study is that the CHIQ is a short, CH-specific, reliable and valid measure of CH-related disability. Therefore, the version presented in Additional file 1 is to be regarded as the final, validated version. To the best of our knowledge, this is the first evaluation of a CH-specific disability questionnaire.

### Reliability and validity of the CHIQ

The CHIQ shows good item statistics, internal consistency and retest-reliability. Of the eight items, item 7 (asking for self-injurious behavior) showed the lowest average score and the weakest (although still adequate) values for item selectivity, item-scale correlation and factor loading. This is likely due to the fact that only part of the patients exhibit self-injurious behavior. However, self-injurious behavior is much more frequent in CH compared to other primary headaches [24] and if present, constitutes a considerable burden to the patient. Therefore, we decided to keep this item.

The CHIQ showed significant positive correlations with attack frequency and acute medication intake frequency during the last week, as well as cluster headache pain AUC, demonstrating that it captures current CH severity. Positive correlations were also found between the CHIQ and the HIT-6, suggesting that the CHIQ also evaluates unspecific headache-related disability. Moreover, the CHIQ was also positively correlated with the DASS depression, anxiety and stress scores and negatively correlated with the SF12v2 PCS and MCS, showing the expected relations to psychosocial factors and QoL.

### Disability in cluster headache

Previous studies have used non-CH-specific questionnaires like HIT-6, MIDAS, HDI or MSQ 2.1 to measure disability in CH patients [3, 4]. These studies have shown higher impairment in chronic and active episodic CH patients compared to eCH patients in remission or healthy controls [25–27]. However, these questionnaires were not designed and validated for CH and may underestimate CH-related disability [5]. On the one hand, they might miss specific CH features such as nocturnal attacks and self-injurious behavior. On the other hand, items asking for the frequency of severe headache days, the desire to lie down or muscle tension due to headache may not be appropriate. Further, these questionnaires use timeframes from four weeks (HIT-6, MSQ 2.1) to three months (MIDAS) or even no timeframe (HDI). CH often shows rapid changes in attack frequency, so shorter timeframes are necessary to capture current impact.

The lack of CH-specific instruments has recently prompted the development of two comprehensive questionnaires, the 28-item Cluster Headache Quality of Life Scale (CHQ) [5] and the 36-item Cluster Headache Scales (CHS) assessing several CH-related psychological factors [6]. The CHS also comprises an 11-item ‘disability’ subscale, that however focuses on generic, not on CH specific questions (e.g. “I feel limited in everyday life” or “I can practice my hobbies”) [6]. Significantly lower QoL was detected using the CHQ in chronic compared to episodic CH patients, but no distinction was made between active and in remission eCH patients [5]. The CHS showed - among others - higher scores in the subscale ‘disability’ in cCH compared to eCH patients who scored higher in the subscale “fear of attacks” [6]. Together, this suggests a higher burden of cCH compared to eCH patients and provides important detail information.

Two further studies evaluated CH-dependent burden without the use of specific questionnaires in 1165 patients (cCH *n* = 306) [28], respectively 1134 patients [29]. Chronic CH patients reported significantly more interictal symptoms [28]. In the other study, 55% of patients reported suicidal ideation and 50% showed self-injurious behavior during attacks [29], emphasizing the need to evaluate self-injurious behavior or suicidal thoughts.

There are also measures that try to assess burden of disease by simply scoring attack frequency, attack duration and duration of episodes, like the Cluster Headache Severity Scale (CHSS) or the CH index [30, 31], an approach that captures only one determinant of disability. To complement the existing measures by a short, CH-specific disability questionnaire, the CHIQ (1) was limited to 8 items, (2) was designed to capture CH-specific characteristics like the impact of nocturnal attacks, the unpredictability of attacks and self-injuring behavior and (3) uses a time frame of 1 week. That makes the CHIQ useful as a short, economic, CH-specific measure of disability in both clinical and research settings. In contrast, if the goal is to plan individual behavioral interventions, the more extensive CHS seems more appropriate.

### CHIQ as a measure of disability

The CHIQ shows correlations with measures of unspecific headache-related disability (HIT-6), with depression, anxiety and stress scores (DASS) and with a generic QoL instrument (SF-12v2). In addition, the CHIQ correlates with CH attack and medication intake frequency, and is rated by patients as appropriate to capture disability related to CH. Moreover, active patients (cCH and active eCH) showed significantly higher CHIQ scores compared to eCH patients in remission, as expected. The CHIQ also captured a higher disability in cCH compared to active eCH, which would be expected due to the chronic nature of cCH, with ongoing attacks without remission. In addition, cCH patients also had a higher attack frequency than active eCH patients in the present study.

In contrast, previous studies using the HIT-6 did not provide a clear picture: one study reported significantly higher HIT-6 scores in cCH compared to active eCH [32], others couldn’t find this difference [27, 33]. In a recent study with 224 CH patients (eCH 70.5%) no difference could be shown for HIT-6 scores between eCH and cCH, maybe due to a rather small cCH group [33]. Also, our study revealed no difference in HIT-6 scores between cCH and active eCH patients.

Interestingly, disability as assessed by the CHIQ was not equal to zero in eCH patients in remission, but relevant disability persisted in spite of cessation of the attacks. This is consistent with previous studies [26] and might reflect a general burden of the disease, e.g. by its long-term psychosocial consequences, the fear of upcoming active headache phases or the knowledge about the disorder itself.

We also propose a preliminary CHIQ grading (grades 1 to 4) based on quartiles of our active CH patient population. This approach has the advantage of generating grades that allow differentiation over the range of observed values. Analysis of attack and medication frequency and other questionnaire scores between grades showed that the proposed CHIQ grades represent a meaningful ordinal scale. In addition, most of the eCH patients in remission fell into grade 1. Nonetheless, for the time being we refrain from assigning clinical attributes (like mild, moderate, severe disability) to CHIQ grades because no existing CH disability scale could be used for comparison. We currently plan a follow-up study including a verbal rating scale on CH disability to help us labelling (and confirming or adjusting) the preliminary CHIQ grades.

### Comparison of CHIQ and HIT-6

Compared to the HIT-6, the CHIQ received better suitability ratings from participants, showed larger correlations with clinical CH characteristics (attack and acute medication frequency and CH pain AUC) and detected a difference between cCH and active eCH patients that was not detected by the HIT-6. This suggests that the CH-specific measure CHIQ better reflects CH-related disability than unspecific measures such as the HIT-6.

### CHIQ as a patient-reported outcome measure

Patient-reported outcome measures (PROMs) become increasingly important in clinical practice and research and complement more objective measures such as number of attacks. They provide a patient-centered approach and are already well implemented in other primary headaches like migraine [8]. PROMs might better reflect patients’ evaluations (like change in QoL) towards a treatment than objective measurements like attack frequency or intake of acute medication [7]. In our sample, this is supported by the fact that also eCH patients in remission showed some disability in spite of having no attacks.

### Strengths and limitations

One strength of the current analysis is the large CH population examined (254 total, 196 patients with active CH). 43.7% of patients had chronic CH, which is more than expected from epidemiological studies [2], likely because these patients more frequently seek medical care and participate in support organizations. Encouragingly, a substantial amount of patients were female (34.3%). In recent decades male preponderance has decreased and female patients are increasingly recognized. An US cluster headache survey showed a female proportion of 28% among 1134 CH patients [34], similar to our study.

One limitation might be the self-reported diagnosis of patients participating via the support group. However, we assessed all criteria necessary to confirm a CH diagnosis according to the ICHD-3 in the survey and included only patients who fulfilled these criteria. Further, 80% and 9% of the patients indicated to have been diagnosed by a neurologist or pain therapist, respectively. Patients were recruited in part from our tertiary outpatient headache clinic and in part via the German CH support group organization (CSG), which might have led to an overrepresentation of severely affected patients. Therefore, results might not be directly generalized to the total population of CH patients. Discriminant validity was not investigated in the present study because we decided against including another (unrelated) questionnaire in the already long survey. Responsiveness could not be evaluated in the current setting due to small patient numbers. However, this is a goal for a further study.

## Conclusions

The CHIQ has a couple of advantages making it a convenient measure of CH-related disability in both clinical routine and research. First, the questionnaire is short, easy to administer and easy to score. This enhances patient compliance and the quality of the captured data. Second, the one week timeframe reduces recall bias and helps assessing the current impact of CH in patients with often rapidly changing attack frequencies. Third, the CHIQ includes CH-specific items and is validated for the use in CH patients. Fourth, the CHIQ is a self-report questionnaire. In clinical routine, patients may be asked to fill the questionnaire immediately before their appointment, giving the physician individual information about current burden and therapeutic needs.

## Supplementary Information

**Additional file 1: Table 1**: The English version of the Cluster Headache Impact Questionnaire (CHIQ). Each question is scored on a Likert scale from 0 = ‘never’ to 5 = ‘always’. The CHIQ score is calculated as the sum of the 8 items. Higher scores indicate higher disability. Two extra questions assess attack frequency and intake of acute medication in the last week. These questions are not part of the CHIQ score. The original German version of the CHIQ can be obtained from the authors.

**Additional file 2: Supplementary Figure 1**: Relation between CHIQ grades and attack frequency, acute medication frequency, pain AUC and results of HIT-6 and DASS. Error bars indicate SEM. Kruskal-Wallis ANOVA for comparison between CHIQ grades was significant at *p* < 0.001 for all measures. AUC, area under the curve. SEM, standard error of the mean. HIT-6, Headache Impact Test; DASS, Depression, anxiety and stress scale.

## Data Availability

The datasets used and analyzed during the current study are available from the corresponding author on reasonable request.

## Abbreviations

BP: Bodily pain
CAS: Cranio-autonomic symptoms
CH: Cluster headache
cCH: Chronic cluster headache
CHIQ: Cluster Headache Impact Questionnaire
CHQ: Cluster Headache Quality of Life Scale
CHS: Cluster Headache Scales
CHSS: Cluster Headache Severity Scale
CSG: Cluster headache support group
DASS: Depression, Anxiety and Stress Scales
eCH: Episodic cluster headache
GH: General health
HIT-6™: Headache Impact Test™
ICC: Intraclass correlation coefficient
ICHD-3: International Classification of Headache Disorders
MCS: Mental component score
MH: Mental health
MIDAS: Migraine Disability Assessment
MSQ v2.1: Migraine Specific Quality of Life Questionnaire Version 2.1
PCS: Physical component score
PF: Physical functioning
PFA: Principal factor analysis
PROM: Patient-reported outcome measure
QoL: Quality of life
RE: Role emotional
RedCap: Research Electronic Data Capture
RP: Role physical
SF: Social functioning
SF-12v2®: Short Form-12 Health Survey
VT: Vitality
