# Supplementary material for: Cluster Headache Impact Questionnaire (CHIQ) – a short measure of cluster headache related disability
Source: J Headache Pain. 2022 Mar 18;23(1):37. doi: 10.1186/s10194-022-01406-y (PMC8932058; doi:10.1186/s10194-022-01406-y)
Supplement: Supplementary file 1 — Additional file 1: Table 1: The English version of the Cluster Headache Impact Questionnaire (CHIQ). Each question is scored on a Likert scale from 0 = ‘never’ to 5 = ‘always’. The CHIQ score is calculated as the sum of the 8 items. Higher scores indicate higher disability. Two extra questions assess attack frequency and intake of acute medication in the last week. These questions are not part of the CHIQ score. The original German version of the CHIQ can be obtained from the authors. [file 10194_2022_1406_MOESM1_ESM.docx]

**Cluster Headache Impact Questionnaire (CHIQ)**

The aim of this questionnaire is to describe the **CURRENT** impact of cluster headache on your daily life. **Please tick the answer to each question that best describes your condition LAST WEEK.**

1. How often did your headaches have an impact on your ability to work, do housework or meet other responsibilities?

| □ Never | □ Rarely | □ Sometimes | □ Often | □ Very often | □ Always |
| --- | --- | --- | --- | --- | --- |

1. How often did your headaches have an impact on your family life, leisure activities or social contacts?

| □ Never | □ Rarely | □ Sometimes | □ Often | □ Very often | □ Always |
| --- | --- | --- | --- | --- | --- |

1. How often have you felt too tired to work or carry out your daily activities because of headaches at night?

| □ Never | □ Rarely | □ Sometimes | □ Often | □ Very often | □ Always |
| --- | --- | --- | --- | --- | --- |

1. How often were you irritated or fed up with everything because of headaches?

| □ Never | □ Rarely | □ Sometimes | □ Often | □ Very often | □ Always |
| --- | --- | --- | --- | --- | --- |

1. How often were you afraid to plan anything because of the unpredictability of the headache attacks?

| □ Never | □ Rarely | □ Sometimes | □ Often | □ Very often | □ Always |
| --- | --- | --- | --- | --- | --- |

1. How often were you unable to think clearly or concentrate even between the attacks?

| □ Never | □ Rarely | □ Sometimes | □ Often | □ Very often | □ Always |
| --- | --- | --- | --- | --- | --- |

1. How often did you do harmful things to yourself (such as hitting your head or biting the inside of your cheek) when you had a headache?

| □ Never | □ Rarely | □ Sometimes | □ Often | □ Very often | □ Always |
| --- | --- | --- | --- | --- | --- |

1. How often did you feel you were a burden to others because of your headaches?

| □ Never | □ Rarely | □ Sometimes | □ Often | □ Very often | □ Always |
| --- | --- | --- | --- | --- | --- |

How many attacks did you have altogether last week? _____ attacks

How often did you take acute medication for your cluster headaches last week? _____ triptans/ oxygen
