# Supplementary material for: Cluster Headache Impact Questionnaire (CHIQ) – a short measure of cluster headache related disability
Source: J Headache Pain. 2022 Mar 18;23(1):37. doi: 10.1186/s10194-022-01406-y (PMC8932058; doi:10.1186/s10194-022-01406-y)
Supplement: Supplementary file 2 — Additional file 2: Supplementary Figure 1: Relation between CHIQ grades and attack frequency, acute medication frequency, pain AUC and results of HIT-6 and DASS. Error bars indicate SEM. Kruskal-Wallis ANOVA for comparison between CHIQ grades was significant at p < 0.001 for all measures. AUC, area under the curve. SEM, standard error of the mean. HIT-6, Headache Impact Test; DASS, Depression, anxiety and stress scale. [file 10194_2022_1406_MOESM2_ESM.pdf]

Supplementary Fig. 1

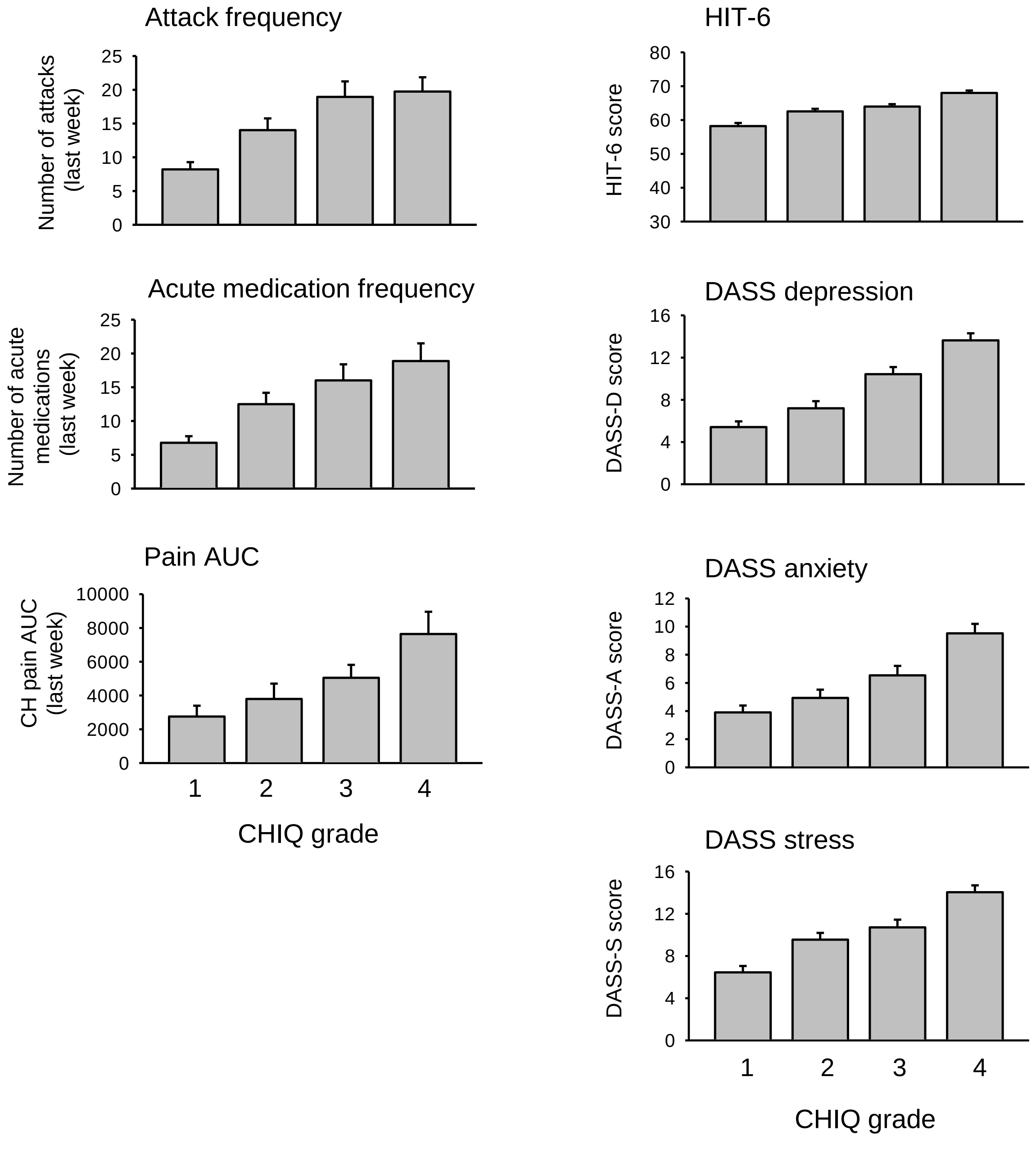

Supplementary Fig. 1: relation between CHIQ grades and attack frequency, acute medication frequency, pain AUC and results of HIT-6 and DASS. Error bars indicate SEM. Kruskal-Wallis ANOVA for comparison between CHIQ grades was significant at p<0.001 for all measures. AUC, area under the curve. SEM, standard error of the mean. HIT-6, Headache Impact Test; DASS, Depression, anxiety and stress scale.
